# Genetic loss-of-function does not support gain-of-function studies suggesting retinoic acid controls limb bud timing and scaling

**DOI:** 10.3389/fcell.2023.1149009

**Published:** 2023-04-13

**Authors:** Marie Berenguer, Gregg Duester

**Affiliations:** Development, Aging, and Regeneration Program, Sanford Burnham Prebys Medical Discovery Institute, La Jolla, CA, United States

**Keywords:** retinoic acid signaling, limb development, genetic loss-of-function, gain-of-function, limb patterning

Retinoic acid (RA) is a potent epigenetic regulator that directly controls transcription of key genes during development. This article addresses concerns about recently published data suggesting that RA influences the timing and scaling of limb development. RA gain-of-function (GOF) studies in chick embryos where RA is added at pharmacological levels were used to conclude that RA signaling controls the timing and scaling of limb development. However, previous RA loss-of-function (LOF) studies in mouse contradict the conclusions derived from chick GOF studies, instead demonstrating that endogenous RA is not required for timing, scaling, and patterning of limbs during development. As genetic LOF studies are generally considered superior to GOF studies for determining the normal functions of genes, proteins, and signaling molecules (such as RA), readers need to be aware that chick studies do not provide conclusive evidence on the normal function of endogenous RA during limb development.

Studies on the mechanisms underlying development of limbs have led to great insight into how epigenetic regulators such as RA control transcription (Cunningham and Duester, 2015). However, studies on how retinoic acid (RA) regulates limb development have been much more controversial. Chick GOF studies have shown that limbs treated with RA have altered proximodistal patterning that forces expression of *Meis1/Meis2* to extend from its normal proximal position to a distal position; this observation led to the conclusion that RA normally controls limb proximodistal patterning by activating *Meis1/Meis2* in the proximal limb (Mercader et al., 2000; Rosello-Diez et al., 2011). In contrast, mouse RA genetic LOF studies show that RA is not required for limb proximodistal patterning; reviewed in (Cunningham and Duester, 2015). The main problem concerning chick RA GOF studies revolves around the fact that RA is a small molecule that can be added at very high micromolar (μM) levels in GOF studies (Mercader et al., 2000; Rosello-Diez et al., 2011) compared to its normal endogenous levels which are nanomolar (nM) in various tissues of both mouse and chick embryos including limb buds that have ∼25 nM RA (Dong and Zile, 1995; Horton and Maden, 1995). In essence, RA added at pharmacological μM levels to embryos or cell lines has side-effects on transcription that allow RA to now control genes that it would not normally control when RA is present at endogenous nM levels (Cunningham and Duester, 2015).

Recently, Stainton and Towers (2022) reported studies using chick embryo limb buds treated with pharmacological levels of RA (GOF) or an RA receptor antagonist (to simulate LOF) along with tissue grafting to make the conclusion that RA normally controls the timing and scaling of limb development. In these studies, beads soaked with high levels of RA [0.05 mg/mL; ∼0.1 millimolar (mM)] or an RAR antagonist (1 mg/mL; ∼2 mM) were implanted in the limb, with the reagent then diffusing into the limb (Stainton and Towers, 2022). Although the limb tissue near the bead may not achieve the extremely high mM concentration of reagent present in the bead, it is clear that the nearby limb tissue is exposed to a much higher concentration than the 25 nM of endogenous RA (probably μM levels), thus resulting in potential off-target effects. Also, since RA receptor antagonists silence genes in the vicinity of RA receptor-bound RA response elements (Germain et al., 2002), a high concentration may dominantly switch off numerous genes that happen to have a RA response element nearby but that normally use other control elements under physiological RA conditions. RA receptor chromatin immunoprecipitation studies have discovered 13,000–15,000 potential RA response elements (Lalevee et al., 2011; Moutier et al., 2012), most of which have not been attributed to endogenous RA signaling, but many of which may become off-targets during treatment with high amounts of RA or RA receptor antagonists (Cunningham and Duester, 2015). Thus, RA receptor antagonists may not provide the intended LOF result.

In contrast to these chick studies (Stainton and Towers, 2022), several previously published genetic LOF studies describing RA-deficient mouse embryos all contradict their main conclusion (Sandell et al., 2007; Zhao et al., 2009; Cunningham et al., 2011; Cunningham et al., 2013; Berenguer et al., 2020). Our questioning of the conclusions made by Stainton and Towers (2022) is reinforced by several review articles indicating that RA is not required for limb proximodistal patterning (Lewandoski and Mackem, 2009; Kawakami, 2013; Cunningham and Duester, 2015; Ghyselinck and Duester, 2019; Berenguer and Duester, 2021). Stainton and Towers (2022) reference only one old paper related to mouse RA LOF studies, (Mic et al., 2004), in order to support the view that RA controls limb proximodistal patterning, without referencing more recent mouse papers that reject this idea. Mic et al. (2004) partially supported the limb RA proximodistal hypothesis by showing that RA signaling activity is indeed present in proximal but not distal tissue of mouse limb buds; the proximal limb does not generate RA itself but instead receives RA by diffusion from the adjacent trunk that expresses RA-generating enzymes. Mic et al. (2004) also analyzed mouse knockout embryos for *Aldh1a2* encoding retinaldehyde dehydrogenase 2 (ALDH1A2; RALDH2) that performs the final step of RA synthesis (retinaldehyde to RA); however, *Aldh1a2*−/− embryos were not able to verify the RA limb proximodistal hypothesis as these embryos completely lack trunk RA and undergo lethality prior to limb bud formation. Subsequently, an article was published in 2007 by Sandell et al. (2007) who generated a mouse mutant for *Rdh10* encoding retinol dehydrogenase 10 (RDH10) that performs the first step of RA synthesis (retinol to retinaldehyde); *Rdh10* mutant embryos survive long enough to develop limbs because they retain a small amount of trunk RA activity (probably due to another unknown retinol-metabolizing enzyme), but limb buds do not exhibit RA signaling activity and proximodistal patterning of hindlimbs is normal whereas forelimbs are stunted. Interestingly, a recent genetic LOF study shows that conditional incomplete loss of all three mouse RA receptors results in embryos with a stunted forelimb but a hindlimb of normal size similar to *Rdh10* mutants (Teletin et al., 2023). These mouse LOF studies suggest that loss of RA signaling does not effect the timing and scaling of limb buds, especially hindlimb buds.

Further studies on the mouse *Rdh10* knockout showed that RA is not required for limb proximodistal patterning, but that RA is required for initiation of forelimb budding through repression of the gene encoding fibroblast growth factor 8 (*Fgf8*) in the forelimb field (Zhao et al., 2009; Cunningham et al., 2013); *Fgf8* is normally expressed anteriorly in the heart and posteriorly in the tail bud progenitors, but if FGF8 is present between these domains in the trunk region where the forelimb field lies, FGF8 will inhibit expression of *Tbx5* that is required to initiate forelimb budding (Zhao et al., 2009; Cunningham et al., 2013). Although loss of RA inhibits initiation of forelimb budding, these more recent papers all show that hindlimbs grow normally in the complete absence of RA and the stunted forelimbs as well as the normally-sized hindlimbs both have normal proximodistal patterning, i.e., they still exhibit a proximal domain with *Meis1/Meis2* expression and a distal domain with *Hoxa11* expression (Zhao et al., 2009; Cunningham et al., 2011; Cunningham et al., 2013). Some concerns have been raised that maybe RA signaling has not been efficiently removed from limbs of *Rdh10* mutants, however our analysis of RA levels demonstrated that the RA concentration in mouse limb buds, which is normally about 25 nM (Horton and Maden, 1995), was easily detected in wild-type but undetectable in *Rdh10* mutant limbs with the results showing a reduction of at least 100-fold to less than 0.25 nM in both forelimbs and hindlimbs (Cunningham et al., 2013). Thus, contrary to the conclusions of Stainton and Towers (2022) based on chick GOF studies, mouse genetic LOF studies show that endogenous RA is not required for patterning of forelimbs and hindlimbs (Zhao et al., 2009; Cunningham et al., 2013). In addition, the mouse studies show that loss of RA has no effect on timing and scaling of hindlimbs, and forelimb timing is not changed by loss of RA in *Rdh10* mutants while the stunted forelimb is best described as a toxic effect of excess trunk/heart FGF8 signaling diffusing into the forelimb field prior to outgrowth rather than an effect on scaling (Zhao et al., 2009; Cunningham et al., 2013). As Stainton and Towers (2022) examined only forelimbs (wings), one might say that their results on timing and scaling do not apply to hindlimbs (legs). However, it is reasonable to assume that their conclusions would also apply to hindlimbs as the original hypothesis stated that both forelimbs and hindlimbs use RA to control limb proximodistal patterning (Mercader et al., 2000). As the mouse LOF data very clearly show that complete loss of RA signaling has no effect on hindlimb size, timing, or patterning, this raises concerns that the original chick conclusion (Mercader et al., 2000) and subsequent studies (Cooper et al., 2011; Rosello-Diez et al., 2011; Stainton and Towers, 2022) are based on side-effects of pharmacological RA treatments and grafting experiments rather than an essential role of endogenous RA. One can conclude that a role for endogenous RA in limb size, timing, and patterning does not exist in mice. Although the conclusion made from chick limb studies have been suggested to apply to vertebrates in general (Mercader et al., 2000; Cooper et al., 2011; Rosello-Diez et al., 2011), a role for endogenous RA might exist in the chick limb but not all vertebrate limbs. However, a chick genetic LOF study would be needed to confirm that RA is required in the chick limb.

A mouse knockout of *Cyp26b1*, encoding an RA-degrading enzyme expressed in the distal limb, exhibits stunted limbs and ectopic expression of *Meis1/Meis2* in the distal limb; these observations were suggested to support the hypothesis that RA is required for limb proximodistal patterning, with CYP26B1 setting a border where RA can activate *Meis1/Meis2* proximally but not distally (Yashiro et al., 2004). In the *Cyp26b1* knockout, although RA activity and *Meis1/Meis2* expression expand into the distal limb, similar to chick RA-treatment studies, limbs exhibit proximal as well as distal truncation likely due to higher than normal RA activity in the proximal limb as well as RA distally (Yashiro et al., 2004); these observations are inconsistent with RA functioning to induce proximal identity. In our opinion, the *Cyp26b1* knockout results in a teratogenic phenotype similar to embryos treated with exogenous RA that exhibit increased apoptosis and a block in chondrogenesis along the entire axis of the limb (Pennimpede et al., 2010; Dranse et al., 2011). Thus, *Cyp26b1* expression distally may not function to restrict RA signaling proximally to set the boundary of *Meis1/Meis2* expression for proximodistal patterning, but instead *Cyp26b1* may function to simply eliminate RA activity distally as a sink to reduce RA all along the limb axis to prevent teratogenesis.

Studies on a conditional mouse *Meis1/Meis2* double knockout that reduces expression confirmed that *Meis1/Meis2* functions as a proximal signal needed to control limb proximodistal patterning, and that normal expression of these genes is prevented distally by FGF8 signals from the apical ectodermal ridge (Delgado et al., 2020). Further studies demonstrated that a complete *Meis1/Meis2* double knockout fails to initiate forelimb development, showing that *Meis1/Meis2* are required not only for limb proximodistal patterning but also for initiation of forelimb buds (Berenguer et al., 2020). Combined with studies showing that the mouse *Rdh10* knockout (which lacks RA signaling in limb buds) still expresses *Meis1/Meis2* in forelimbs and hindlimbs, the original hypothesis for control of limb proximodistal patterning (Mercader et al., 2000) needs revision. A revised hypothesis proposes the following: (a) trunk RA signaling acts permissively to allow limb bud initiation by directly repressing *Fgf8* in the limb field to separate it from *Fgf8* expression domains anteriorly in the heart and posteriorly in the tail bud where the body axis is extending; (b) *Meis1/Meis2* expressed in trunk mesoderm fated to become the limb field functions instructively along with *Tbx5* to initiate limb budding; (c) after limb outgrowth begins, *Meis1/Meis2* expression is activated in proximal limb by a signal other than RA; (d) *Meis1/Meis2* expression is limited to the proximal limb due to expression of *Fgf8* distally in the apical ectodermal ridge; (e) *Meis1/Meis2* but not RA then functions to control limb proximodistal patterning (Berenguer and Duester, 2021).

In conclusion, genetic LOF studies are needed to make major conclusion on the function of endogenous RA. Even though LOF studies can be difficult, they are required to determine the normal function of any molecule, protein, or gene as GOF studies (such as treatments with small molecules or proteins as well as overexpression of genes) may lead to side-effects that prevent insight into how endogenous signaling systems function.
